# Protective effects of *Cornus mas* fruit extract on methotrexate-induced alterations in mice testicular tissue: Evidences for histochemical and histomorphometrical changes in an animal model study

**DOI:** 10.30466/vrf.2019.69516.1955

**Published:** 2019-12-15

**Authors:** Liela Zarei, Rasoul Shahrooz

**Affiliations:** 1 *Department of Anatomical Sciences, Faculty of Medicine and Razi Herbal Medicines Research Center, Lorestan University of Medical Sciences, Khorramabad, Iran* *;*; 2 *Department of Basic Sciences, Faculty of Veterinary Medicine, Urmia University, Urmia, Iran.*

**Keywords:** Cornus mas, Histochemistry, Methotrexate, Mice, Testis

## Abstract

Methotrexate (MTX) as a chemotherapeutic agent, has adverse effects on reproductive organs by enhancing oxidative stress. In this study, the protective effects of *Cornus mas* fruit extract (CMFE) against MTX side effects were evaluated. Forty-eight mature male NMRI mice were divided into six groups: group 1 (control) received 0.10 mL per day of normal saline intraperitoneally (IP), group 2 received MTX (20.00 mg kg^-1^ per week, IP), group 3 received MTX along with CMFE 250 mg kg^-1^ per day by oral gavage, group 4 received MTX along with CMFE 500 mg kg^-1^ per day by oral gavage, group 5 received MTX plus 1000 mg kg^-1 ^per day of CMFE by oral gavage, and group 6 received 1000 mg kg^-1^ per day of CMFE extract, orally. All animals were treated for 35 consecutive days. Thickness of testicular capsule and germinal epithelium and diameter of seminiferous tubules were measured. Intra-cytoplasmic levels of carbohydrate, unsaturated fatty acid (UFA) and alkaline phosphatase were assessed. Serum level of testosterone and testicular total antioxidant capacity (TAC) were also evaluated. The results demonstrated that MTX administration caused morphometrical parameters except the thickness of testicular capsule were significantly different in comparison to control group and decreased cytoplasmic concentration of carbohydrate in the first three layers of germinal epithelium and increased the UFA levels. Contrarily, CMFE ameliorates the condition. Moreover, CMFE increased testosterone level and increased the MTX-reduced TAC level. In conclusion, it was revealed that CMFE decreased the cellular atrophy by controlling the energy substrate utilization based on lipids and carbohydrates via provoking the testicular antioxidant status.

## Introduction

Methotrexate (MTX), a folate antagonist compound, was introduced in 1950 as a chemotherapeutic drug.^1^ It is used to control the tumor malignancies including acute and chronic leukemia, lymphoma, bladder cancer, breast cancer, and testicular tumors.^2^^,^^3^ Moreover, MTX is administrated as an immunosuppressive drug in patients with autoimmune disorders like arthritis rheumatoid and psoriasis. ^4^^,^^5^ A study has shown that MTX could induce degenerative changes in the reproductive system of male Swiss mice.^6^ It was also reported that the administration of MTX resulted in a significant reduction in sperm count, an increase in sperm morphological disorders, and sperm DNA disintegration, all associated with the testicular damages caused by MTX.^7^

It has been indicated that the administration of 25.00 mg kg^-1^ and 50.00 mg kg^-1^ of MTX in adult rats resulted in elevated damages to seminiferous tubules and increased edema of the interstitial tissue of the testes.^8^ It was shown that intramuscular administration of 0.15 and 0.30 mg kg^-1^ of MTX in both male and female adult mice remarkably reduced spermatogenesis index and follicular growth of ovarian tissue, respectively.^9^

The oxidative stress induced by MTX was considered as the main mechanism for the degenerative impacts of this chemical agent in testicular and brain tissues.^10^^-^^12^ This oxidative stress in testicular tissue correlates indirectly with the lowered endocrine status.^8^^-^^10^^,^^12^ Accordingly, diminished Leydig cells distribution, as well as the lowered testosterone levels in serum, are associated with down-regulated antioxidant status in MTX-administrated rats. This clarifies the correlation between defected endocrine activities and enhanced oxidative stress following chronic exposure to MTX.^8^^,^^9^

*Cornus mas* fruit (CMF)*,* commonly known as cornelian cherry, is a deciduous fruit of a shrub or small tree, native to central and southern Europe and western Asia.^13^ In Iranian traditional herbal therapy, CMF is used to control inflammation, stomach aches and cramps, diarrhea, different skin infections, intestinal parasites, and hemorrhoids.^14^^-^^16^ Biochemical analysis of CMF has shown that it is a rich resource of anti-oxidant and phenolic compounds. In addition, it contains some vitamins including vitamin C, B1, B2, E, as well as anthocyanins, flavonoids, along with very high levels of oxalic acid.^15^^,^^16^ Considering the antioxidant and anti-inflammatory properties of CMF, the present study was designed to evaluate the protective effect of CMFE on MTX-induced damages to testicular tissue and sperm levels. For this purpose, the histochemical analyzes were carried out for detecting any changes in alkaline phosphatase (ALP) enzyme synthesis rate, and intra-cytoplasmic carbohydrate and unsaturated fatty acid ratios. Biochemical analyses for measuring the testicular antioxidant capacity (TAC) and serum level of testosterone were also carried out.

## Materials and Methods

**Animals. **Forty-eight mature healthy male NMRI mice were included in the present study. All animals were kept for one week in standard conditions including temperature 22.00 ± 2.00 ˚C, relative humidity (30.00 to 60.00%) and light/dark cycle (10hr/14hr) in the animal house of Faculty of Veterinary Medicine, Urmia University and fed with standard pellet diet and water. 

The study protocol was reviewed and approved by Urmia University Ethical Committee (AECVU-154-2018). All animals received humane care following the “Guide for the Care and Use of Laboratory Animals” prepared by the “National Academy of Sciences” and published by the “National Institutes of Health” (NIH).^17^

**Preparation of hydro-alcoholic extract of** ***Cornus mas*** **fruit extract (CMFE). **Fresh CMFs were collected from the rural regions of Qazvin province, Iran and identified by a botanical expert, at the Department of Botanic Sciences, Hamadan Research Center for Agri-cultural and Natural Resources, Hamadan, Iran. 

The fruits were dried naturally in room temperature (23.00 - 24.00 ˚C) for six days and pulverized to powder using an electric blender. Then, 100 g of the plant powder was suspended in 600 mL of 50/50% (v/v) hydro- ethanolic solution for 96 hr at room temperature. The mixture was then filtered using a fine muslin cloth followed by a filter paper (Whatman No 1). The filtrate was placed at 40.00 ˚C in an oven to dry. The obtained clear extract was used in the study. The extract was kept at – 15.00 ˚C until it was used in the experiment.^16^

**Study design. **Frothy-eight mature male NMRI mice were randomly divided into six groups of eight mice in each group as follows: 1) Control group which received 0.10 mL per day of normal saline intraperitoneally (IP), 2) MTX group (Koçak Farma, Tekirdağ, Turkey) which received 20.00 mg kg^-1^ per week of MTX, IP, 3) MTX/CMFE/250 group which received MTX in the same way of group 2 plus 250 mg kg^-1^ per day of CMFE orally via oral gavage, 4) MTX/CMFE/500 group which received MTX plus 500 mg kg^-1 ^per day of CMFE via oral gavage, 5) MTX/CMFE/1000 group which received MTX plus 1000 mg kg^-1^ per day of CMFE via oral gavage, and 6) CMFE group which received 1000 mg kg^-1^ per day of CMFE extract via oral gavage. All animals were treated daily for 35 consecutive days.^18^

**Gas chromatography-mass spectrometry (GC-MS) analysis of CMFE. **The GC-MS was equipped with an HP-5MS (30 m × 0.25 mm, 0.25 μm film thickness; Hewlett-Packard, Palo Alto, USA) non-polar capillary column. The Column temperature was set initially at 50.00 ˚C for 1 min, then gradually increased to 220 ˚C with a temperature ramp of 4.00 ˚C per min and finally held at 220˚C for 5 min. The carrier gas was helium at a flow rate of 2.94 mL min^-1^. The injector temperature was set at 240 ˚C employing the split injection mode and the split ratio of 1:50. The MS scan conditions were: Ion source temperature: 200 ˚C; interface temperature: 250 ˚C; ionization energy: 70.00 eV; mass scan range: 40.00 - 350 m/z.

**Histological analyses. **After five weeks, all animals were anesthetized by IP administration of ketamine (90.00 mg kg^-1^; Alfasan, Woerden, Holland) and xylazine (5.00 mg kg^-1^; Alfasan), then one half of the right and left testes were fixed in 10.00% neutral buffered formalin and after tissue processing, embedded in paraffin. Semi-sections (5.00 µm) were stained by hematoxylin and eosin dyes for histomorphometric analyses. The other half of the right and left testes were fixed for histochemical studies. The histomorphometric parameters (capsular thickness, seminiferous tubules diameter and height of germinal epithelium) were measured using a light microscope (Olympus, Tokyo, Japan) with calibrated graded optical lens. Each grade that observed at 400×, estimated 2.55 µm or 100× magnification estimated 10.89 µm.

**Histochemical examination of testicular alkaline phosphatase (ALP) activity. **Histochemical measurement of ALP was performed using a modified method described previously.^19 ^Briefly, tissue sections were deparaffinized in xylene and hydrated through graded descending alcohol series, and incubated for overnight in a solution of 1.00% magnesium chloride in 100 mm Tris maleate buffer (pH 9.20; Sigma, St. Louis, USA). Tissue sections were then incubated for 2 hr at room temperature in a solution containing ALP substrate, freshly prepared 100 mM Tris-maleate buffer (pH 9.20), 0.20 mg mL^-1^ naphthol AS-MX phosphate (Sigma) and 0.40 mg mL^-1^ Fast Red TR. After washing with distilled water, the sections were counterstained with hematoxylin and mounted with Kaiser’s glycerol jelly (Merck, Darmstadt, Germany).

**The assessment of intra-cytoplasmic carbohydrate amount. **To evaluate the carbohydrate amount, a periotic Acid Schiff (PAS) kit (Pajohesh Asia, Tehran, Iran) was used. In brief, the paraffin sectioned specimens were deparaffinized and rehydrated. Hydrated slides were oxidized in 5.00% of the periodic acid solution for 5 min. Having rinsed in distilled water, the slides were placed in Schiff reagent for 15 min and washed with lukewarm water. After 5 min of incubation in room temperature, the slides were counterstained with Meyer’s hematoxylin.

**Unsaturated fatty acid (UFA) analyses. **The Oil Red O staining special commercial kit was used for UFA measurement based on manufacturer instructions (Ayandeh Science Co. Urmia, Iran). Briefly, samples that were fixed in 10.00% neutral buffered formalin were sectioned in frozen by cryostat microtome (Bright Instrument Co. Ltd., Huntingdon, UK) to 10.00 µm slices at air-dried conditions. Then, the slides were fixed again in formalin and thoroughly washed with distilled water before being rinsed in 60.00% isopropanol. Then, the slides were stained with freshly prepared Oil Red O solution for 15 min. The slides were rinsed again in 60% isopropanol and lightly stained with hematoxylin. Finally, the slides were rinsed several times with distilled water and mounted with glycerol jelly. 

**Blood sampling and hormonal assay. **Blood samples were collected from the mice and serum were separated by centrifugation at 3000 *g* for 5 min. Testosterone was measured by an immuneradiometric method using WHO/Sigma Asso-RTGC-768/98 kit (Abbott Laboratories, Abbott Park, USA). The intra-assay coefficient of variance and inter-assay coefficient of variance of 10 tests for testosterone were 3.56 and 8.98, respectively. 

**Assessment of testicular TAC level. **An amount of 0.30 - 0.40 g of the testicular tissue was homogenized in ice-cold KCl (150 mM) and centrifuged at 3000 *g* for 10 min. The supernatants were used for evaluating TAC level. The assessment of TAC was carried out based on the ferric reduction antioxidant power (FRAP) assay as described previously.^20^

**Statistical analyses. **All the histomorphometrical, hormonal and TAC data were expressed as mean ± SD. Data were statistically analyzed using SPSS software (version 20.0; IBM Corp. Armonk, USA) using one-way ANOVA followed by Bonferroni post-hoc test. A *p*-value less than 0.05 was considered significant.

## Results

**GC-MS findings. **GC-MS analysis of the CMFE by HP-5MS column revealed the presence of 108 components. The CMFE consisted of 34.26% alcohols (17.59% aliphatic, 8.33% monoterpenic, 5.56% sesquiterpenic, and 2.78% aromatic alcohols), 22.23% hydrocarbons (12.04% ali-phatic, 1.85% aromatic, 7.41% sesquiterpenic, and 0.93% diterpenic hydrocarbons), 11.11% esters (2.78% aliphatic, 2.78% fatty acid methyl, 4.62% fatty acid ethyl, and 0.93% terpenoid esters), 10.19% ketones (7.40% aliphatic ketones, 1.85% lactones, 0.93% benzoquinone derivatives), 8.33% aldehydes (2.78% aliphatic, 3.70% monoterpenic, and 1.85% aromatic aldehydes), 7.41% acids (0.93% aliphatic, and 6.48% fatty acids), and 6.48% heterocyclic compounds (2.78% furan derivatives and 3.70% terpenoids). The major components of the extract of the mature fruits were: 16-kaurene, α-terpineol, 1-tridecene, α-cadinene, 1-octen-3-ol, furfural, and 1- pentadecene. 

**CMFE inhibited the MTX-induced histological damages. **The results demonstrated that MTX caused severe histological degenerations in testes. The animals received MTX, intensive seminiferous tubules atrophy, germinal cells dissociation, and severe edema in the testicular interstitial tissue were observed. However, the administration of CMFE, in a dose-dependent manner, inhibited the MTX-induced damages. Accordingly, the germinal epithelial dissociation in seminiferous tubules, as well as the edema, was reduced in a dose-dependent manner. Comparison of the testis capsular thickness (to check fibrosis) among study groups showed no significant differences. But, the capsular thickness was more in the MTX group in comparison to other groups. Meanwhile, CMFE reduced the capsular fibrosis in a dose-related manner. There were no histological changes in testes of the animals received CMFE (Table 1). 

**Table 1:** Testicular histomorphometric parameters in different experimental groups. The values are expressed as mean ± SD

**Groups**	**Germinal epithelium height (µm)**	**Tubular diameter (µm)**	**Capsule thickness (µm)**
**Control**	50.47 ± 2.50^a^	217.91 ± 2.58^a^	12.35 ± 0.68^a^
**MTX**	30.47 ± 2.58^b^	117.00 ± 2.80^b^	14.71 ± 0.60^a^
**MTX/CMFE/250**	41.75 ± 2.66^a^	175.83 ± 21.98^ac^	12.78 ± 0.57^a^
**MTX/CMFE/500**	42.51 ± 2.23^a^	196.82 ± 3.69^ac^	12.93 ± 0.43^a^
**MTX/CMFE/1000**	41.15 ± 1.00^a^	184.34 ± 8.39^ac^	14.42 ± 0.47^a^
**CMFE**	46.35 ± 2.17^a^	201.46 ± 1.94^ac^	14.63 ± 0.63^a^

MTX: Methotrexate, CMFE: *Cornus mas* fruit extract.

^abc^ Different superscripts in each column are representing significant differences between groups (*p *< 0.05).

**CMFE balanced the intra-cytoplasmic carbohydrate and fatty acid concentrations. **Histochemical analyses showed that MTX in a single administration reversed the cytoplasmic carbohydrate accumulation. Because of the low levels of carbohydrates, the spermatogonia cells and the spermatocytes (types A and B) exhibited faint reaction for PAS staining. Meanwhile, the same cells in the control group animals showed intensive reaction for PAS staining, representing high carbohydrate levels.

**Fig. 1:**
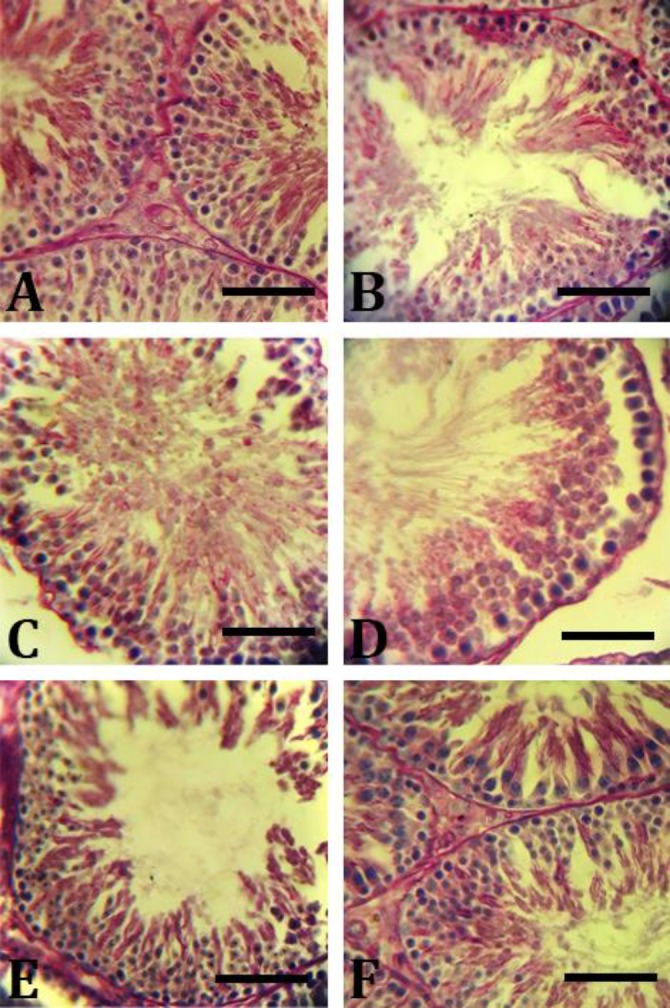
Intra-cytoplasmic carbohydrate amount in testis cross-sections. **A**) Control group, germinal epithelium is presented normally; **B**) MTX group, significant decrease in intra-cytoplasmic carbohydrate amount especially in the first three layers of the germinal epithelium; **C**) MTX/CMFE/250 group; **D**) MTX/CMFE /500 group; **E)** MTX/CMFE/500, co-administration of CMFE enhanced carbohydrate storage in all cellular layers (red reactions) and** F)** MTX/CMFE/1000 group (PAS staining, Bar = 60 µm)

The CMFE and MTX treated animals showed approximately the same staining characteristics to those of the control group. Accordingly, the cytoplasmic carbo-hydrate level was increased in the first three germinal lineages in CMFE treated animals in a dose- dependent manner (Fig. 1). 

Moreover, carbohydrate alterations were calculated in Sertoli and Leydig cells, the results showed that the carbohydrate accumulation was increased in CMFE treated animals versus to MTX group.

**Fig. 2:**
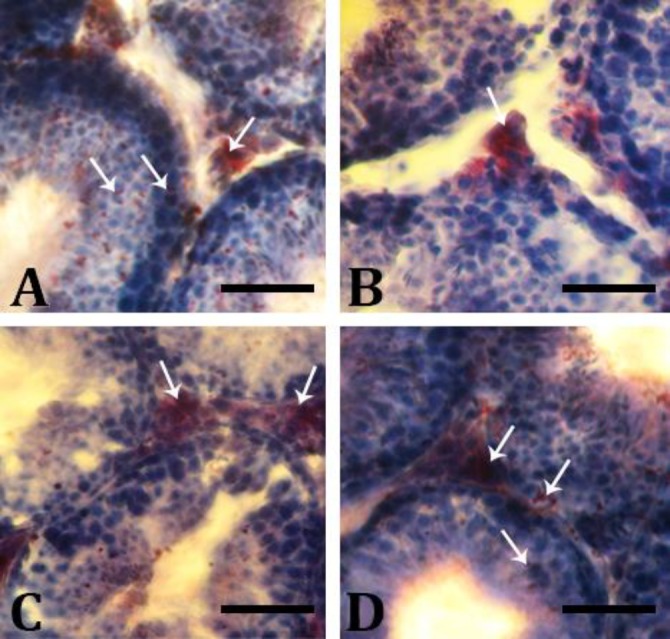
Accumulation of UFA in testis: **A**) Control group: The germinal epithelium of the control testis is presented normally; **B**) MTX group: Decrease in intra-cytoplasmic Oil-Red-O reaction for fatty acids; **C**) MTX/CMFE/500 group; and **D**) MTX/CMFE/ 1000 group. See ameliorated fatty acids accumulation in CMFE group (Oil-Red-O staining, Bar = 60 µm)

Comparison of the intracellular levels of unsaturated fatty acid (UFA) among study groups showed that there was an up-regulation in the UFA concentration in the MTX group in all cellular lineages of the germ layers. The UFA is mainly represented in the spermatid and spermatozoa cell lineages and the first three lineages exhibited faint reactions for Oil Red O staining. In contrast, animals in MTX/MCFE treated groups, with some differences, showed a dense reaction to UFA in the second three lineages of the germ layers and significantly lower reaction in the first three lineages (Fig. 2). The UFA and carbohydrate levels were normal in the CMFE group. 

**Fig. 3:**
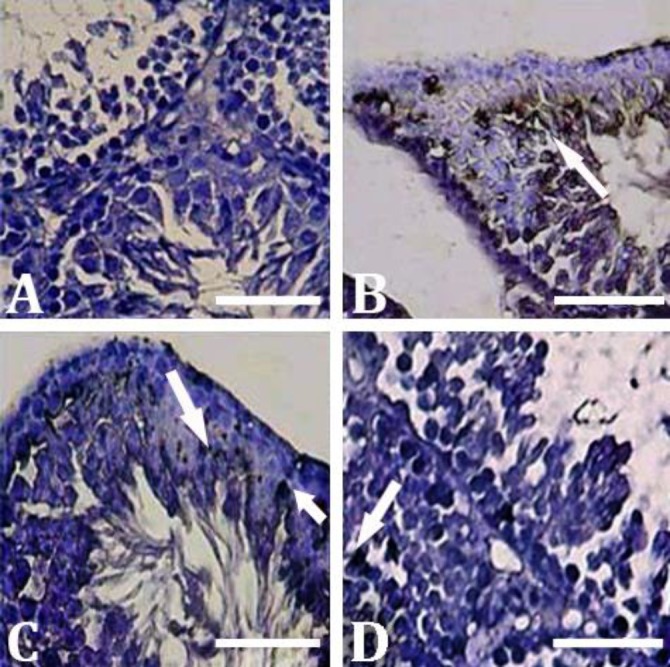
Histochemical analyses for the presence of ALP in testicular tissue. **A)** Control group; **B)** MTX group, intensive increase in ALP activity (arrows); **C)** MTX/CMFE/500 group; and **D)** MTX/CMFE/1000 group. ALP activity was significantly decreased (*p* < 0.05) in CMFE treated animals (Alkaline phosphatase staining, Bar = 60 µm)

**CMFE reduced ALP enzyme activity. **The histo-chemical analyses for cellular ALP activity showed that the animals in the control group exhibited slight ALP activity only in the endothelial cells. However, animals in the MTX group showed significantly higher ALP positive sites in the germ layers, especially in the spermatocytes, spermatids, and Leydig cells compared to the other experimental groups (*p* < 0.05). In contrast, MCFE reduced ALP activity in a dose-dependent manner. Accordingly, MTX/MCF/1000 group showed the lowest ALP activity compared to MTX/MCF/250 and MTX/MCF/500 groups (*p* < 0.05), (Fig. 3). No considerable changes were observed in ALP activity in the CMFE group.

**CMFE increased the serum level of testosterone. **Biochemical assessments revealed that CMFE enhanced the serum level of testosterone in a dose-dependent manner. However, the serum testosterone level was lower in the MTX group compared to the control group (*p* < 0.05). No significant differences were observed in testosterone levels in control and MCFE group animals.

**MCF enhanced the testicular TAC level. **The results demonstrated that CMFE/MTX-treated animals exhibited a significant enhancement in the testicular TAC level. Meanwhile, MTX group showed a significant (*p* < 0.05) reduction in TAC level compared to the control group (*p* < 0.05). No biochemical alterations in TAC level were revealed in CMSE group compared to that of the control group. 

## Discussion

The male gonads are tissues with high rates of mitosis and meiosis and are susceptible to alterations by various anti-tumor chemicals. In the present study, mice were exposed to the various doses of the CMFE to overcome the MTX-induced damages in testis. Our findings indicated that MTX enhanced the inflammation, recognizable by increased ALP synthesis, and changed the energy sources in germinal cell lineages. This was associated with the antioxidant down-regulation which resulted in intensive testicular tissue damages. Moreover, we found that CMFE administration remarkably inhibited the MTX-induced derangements in a dose-dependent manner.

The glucose is considered as the main substrate of energy in the spermatogonia, spermatocytes, and spermatid cell lineages.^21^^,^^22^ *In vitro* experiments demonstrated that the germinal cells use large amounts of lactate and glucose for their mitosis and meiosis.^23^^,^^24^ Carbohydrate staining showed that MTX significantly decreased intra-cytoplasmic carbohydrate concentration especially in Sertoli and spermatogonia cells as well as spermatocyte lineages. Indeed, Sertoli cells metabolize glucose and convert it into lactate. Lactate then secrets from Sertoli cells into the tubular medium via stimulation of the follicular stimulating hormone (FSH).^24^^-^^26^ Therefore, we could conclude that MTX disrupted the glucose and lactate metabolisms, recognizable by decreased intra-cytoplasmic carbohydrate level, and ultimately affected the spermatogenesis process. Meanwhile, the CMFE up-regulated these metabolic pathways, enhanced the cytoplasmic carbohydrate levels, and so raised the MTX-reduced energy substrate in Sertoli and germinal cells. Carbohydrate accumulation in the cytoplasm of Leydig cells was decreased significantly in the MTX group, however, it was reversed in MTX/CMFE groups, dose-dependently. Carbohydrate accumulation in the CMFE group was the same as the control group. 

More intensively, under conditions that the glucose transport and/or metabolism undergoes a severe derangement, the germinal cells switch their energy substrates from glucose to UFA.^21^^,^^27^ Tissue survival and the activity of spermatocytes and spermatids depend strictly on carbohydrate metabolism both at anaerobic and aerobic pathways for maintaining the ATP synthesis.^26^^,^^28^ Therefore, the cells in germinal epithelium use UFAs as a secondary source of energy. Our histochemical analyses showed that all cellular layers exhibited intensive UFA in the animals of the MTX group. However, the MTX and CMFE treated animals, albeit depending on dose, exhibited lower cytoplasmic UFA levels and up-regulated carbo-hydrate accumulation. It seems that CMFE acted by reducing the UFA levels and up-regulated the carbo-hydrate levels which was inhibited by MTX treatment. Sertoli cells in MTX animals exhibited the highest UFA positive foci in the cytoplasm. In order to understand this alteration one should note that Sertoli cells play a critical role in the phagocytosis of residuals during spermato-genesis and spermiogenesis^.^^21^^,^^27^^,^^29^^,^^30^

Our histological analyses showed that the percentage of atrophied seminiferous tubules with germinal epithelium dissociation was increased in the MTX group. In contrast to CMFE animals, the MTX group showed decreased TAC level. The enhancement of abnormal metabolism associated with increased cellular damage and elevated cellular degeneration results in provoking oxidative stress in testicular tissue.^27^^,^^30^ Beside this fact, the down-regulated endocrine status in MTX animals, recognizable by decreased serum level of testosterone, promoted the cellular atrophy and ultimately enhanced the oxidative stress. In fact, Leydig cells control the physiologic interactions of Sertoli cells by secreting testosterone.^31^^,^^32^ Therefore, it would be more logical to conclude that MTX down-regulated the endocrine status and negatively affected Sertoli cells’ involvement in intact spermatogenesis and spermiogenesis processes. This could ultimately elevate cellular degeneration. Due to its high antioxidant activity, CMFE improved survival and secretive potential of Leydig cells and indirectly supported Sertoli cells physiologic function. Higher serum level of testosterone in CMFE animals confirmed this hypothesis. 

The ALP is known as the main marker for inflammation in testicular tissue. Accordingly, the germinal cells, especially dissociated and atrophied ones, exhibited higher levels of ALP than normal cells.^21^^,^^33^ Pathologically-induced inflammation associated with severe cellular damages positively correlates with oxidative stress. Indeed, increased immune cells and inflammation enhance the ROS generation in testicular tissue.^21^^,^^27^^,^^33^ In order to understand the effect of MTX on inflammation and revealing the relation between MTX-induced inflammation and the oxidative stress, we performed ALP staining. Our observations demonstrated that MTX enhanced ALP activity, which is an indicator of intensive inflammation, while CMFE decreased inflammation. However, this study showed that the *Cornus mas* as an antioxidant sometimes maybe has a moderately toxic effect by lowering the level of the testis tissue TAC in healthy animals when administered in a high dose.^34^

The CMFE-treated animals exhibited higher levels of TAC in comparison to the MTX group. It is reasonable to suggest that CMFE could also down-regulate the inflammation due to its antioxidant activity and up-regulate cellular survival. Our findings suggested that CMFE could control energy substrates which were mainly carbohydrates, and inhibit the energy switch into UFA. Moreover, CMFE could improve the testicular endocrine status by enhanced testosterone synthesis. CMFE, as an antioxidant compound, could reduce cellular degeneration, lower inflammation and up-regulate testicular antioxidant status. 
